# Supplementary material for: Identification of the onchocerciasis vector in the Kakoi-Koda focus of the Democratic Republic of Congo
Source: PLoS Negl Trop Dis. 2022 Nov 4;16(11):e0010684. doi: 10.1371/journal.pntd.0010684 (PMC9668120; doi:10.1371/journal.pntd.0010684)
Supplement: S1 Fig — (PDF) [file pntd.0010684.s001.pdf]

PLoSNTDs

Identification of the Onchocerciasis Vector in the Kakoi-Koda Focus of the Democratic Republic of Congo

By Rory J Post, Anne Laudisoit, Christine Laemmer, Kenneth Pfarr, Achim Hoerauf, Michel Mandro, Pablo Tortosa, Yann Gomard, Tony Ukety, Thomson Lakwo, Claude Mande, Lorne Farovitch, Uche Amazigo, Didier Bakajika, David Oguttu, Naomi Awaca & Robert Colebunders

SUPPORTING MATERIAL

S1 Figs: Arial photographs of Kakoi-Koda focus

(Tony Ukety, 20th April 2022)

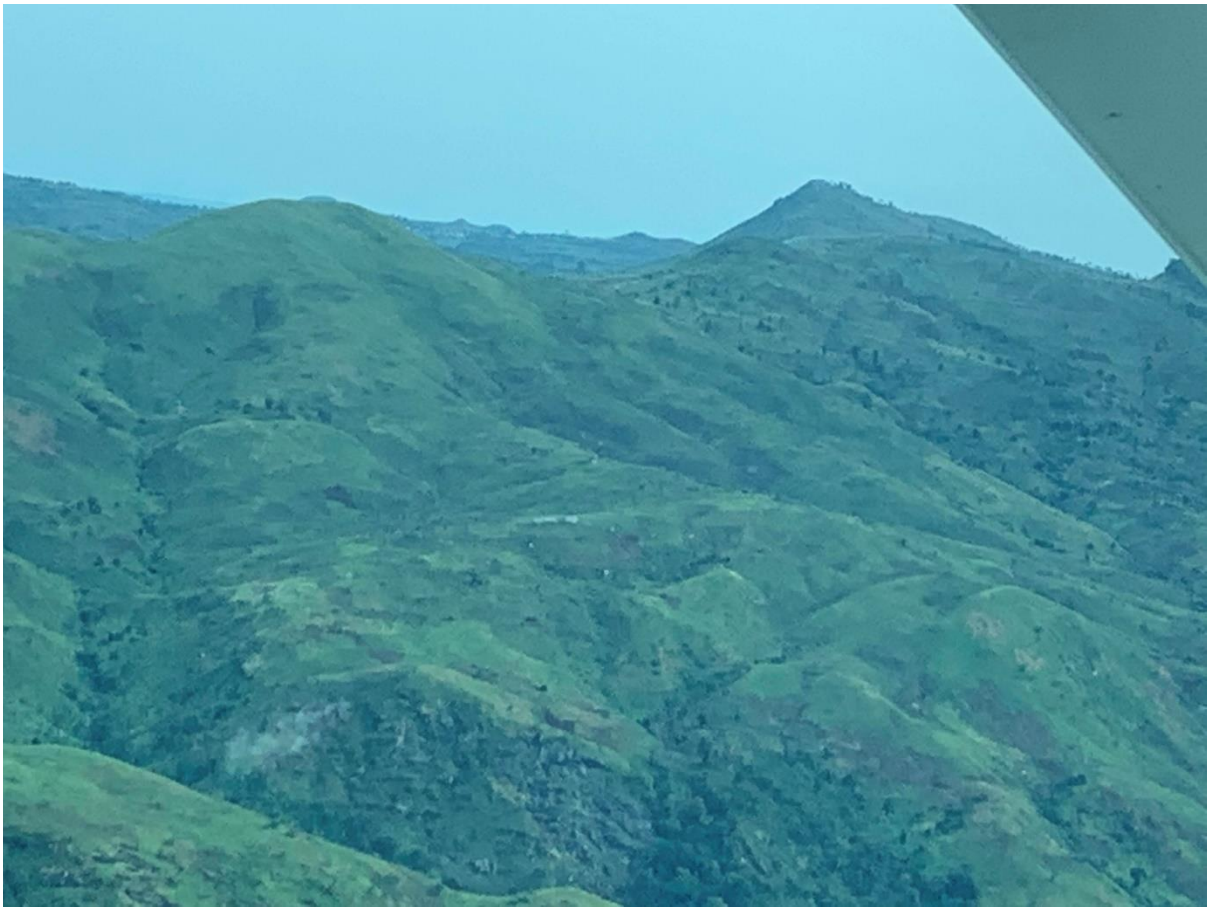

S1a Fig. Mount Aboro viewed from South-East.

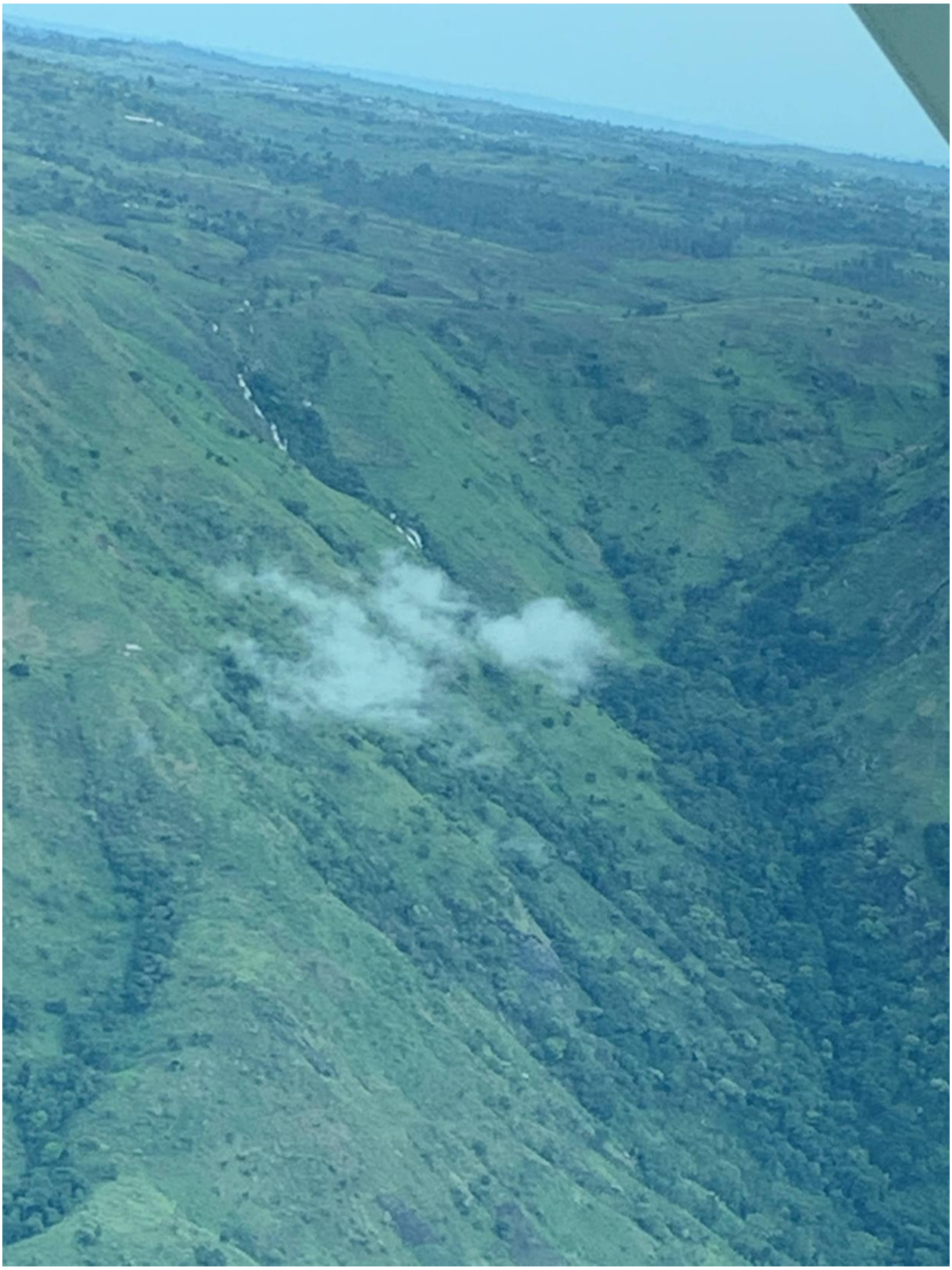

S1b Fig. Koda river viewed from the East.
