# Supplementary material for: Identification of the onchocerciasis vector in the Kakoi-Koda focus of the Democratic Republic of Congo
Source: PLoS Negl Trop Dis. 2022 Nov 4;16(11):e0010684. doi: 10.1371/journal.pntd.0010684 (PMC9668120; doi:10.1371/journal.pntd.0010684)
Supplement: S2 Fig — (available online: https://ecohealthalliance.github.io/KakoiKodaFocus/KakoiKoda_3D_Laudisoit.html). (DOCX) [file pntd.0010684.s002.docx]

**PLoSNTDs**

**Identification of the Onchocerciasis Vector in the Kakoi-Koda Focus of the Democratic Republic of Congo**

By Rory J Post, Anne Laudisoit, Christine Laemmer, Kenneth Pfarr, Achim Hoerauf, Michel Mandro, Pablo Tortosa, Yann Gomard, Tony Ukety, Thomson Lakwo, Claude Mande, Lorne Farovitch, Uche Amazigo, Didier Bakajika, David Oguttu, Naomi Awaca & Robert Colebunders

**SUPPORTING MATERIAL**

**S2 Fig: 3D Relief Map of Kakoi-Koda Focus**

**
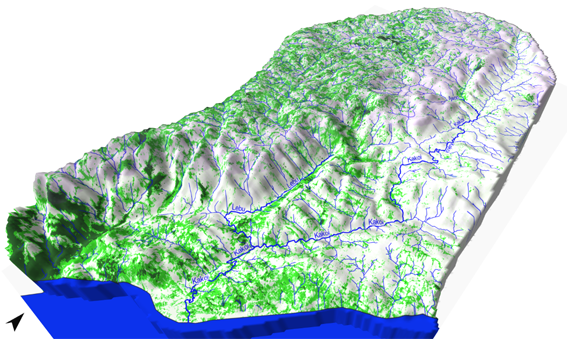
**

The RASTER images used to produce S2 Fig. are Landsat-7 and Landsat-8 images, courtesy of the U.S. Geological Survey (https://www.usgs.gov/)*.* The basic shapefiles for administrative areas can be found on the *Référentiel Géographique Commun* for the DRC (www.rgc.cd).

Note: An interactive version of this map which can be viewed from different scales and angles is available -

<https://ecohealthalliance.github.io/KakoiKodaFocus/KakoiKoda_3D_Laudisoit.html>
