# Supplementary material for: Identification of the onchocerciasis vector in the Kakoi-Koda focus of the Democratic Republic of Congo
Source: PLoS Negl Trop Dis. 2022 Nov 4;16(11):e0010684. doi: 10.1371/journal.pntd.0010684 (PMC9668120; doi:10.1371/journal.pntd.0010684)
Supplement: S1 Table — (PDF) [file pntd.0010684.s003.pdf]

PLoSNTDs

Identification of the Onchocerciasis Vector in the Kakoi-Koda Focus of the Democratic Republic of Congo

By Rory J Post, Anne Laudisoit, Christine Laemmer, Kenneth Pfarr, Achim Hoerauf, Michel Mandro, Pablo Tortosa, Yann Gomard, Tony Ukety, Thomson Lakwo, Claude Mande, Lorne Farovitch, Uche Amazigo, Didier Bakajika, David Oguttu, Naomi Awaca & Robert Colebunders

SUPPORTING MATERIAL

S1 Table: Alphabetical List of Blackfly Species Identified from the Ituri Highlands 2015 to 2018

Genus	Subgenus	Species
Simulium	*Anasolen*	*dentulosum*
	Freemanellum	*berghei*
	Metomphallus	*hargreavesi*
		vorax
	Nevermannia	*bwambanum*
	Pomeroyellum	*alcocki*
		cervicornutum
		impukane/ituriense
		unicornutum

(see S2 Table for details, and S4 Table for *S. neavei*)
