# Supplementary material for: Identification of the onchocerciasis vector in the Kakoi-Koda focus of the Democratic Republic of Congo
Source: PLoS Negl Trop Dis. 2022 Nov 4;16(11):e0010684. doi: 10.1371/journal.pntd.0010684 (PMC9668120; doi:10.1371/journal.pntd.0010684)
Supplement: S2 Table — (PDF) [file pntd.0010684.s004.pdf]

PLoSNTDs

Identification of the Onchocerciasis Vector in the Kakoi-Koda Focus of the Democratic Republic of Congo

By Rory J Post, Anne Laudisoit, Christine Laemmer, Kenneth Pfarr, Achim Hoerauf, Michel Mandro, Pablo Tortosa, Yann Gomard, Tony Ukety, Thomson Lakwo, Claude Mande, Lorne Farovitch, Uche Amazigo, Didier Bakajika, David Oguttu, Naomi Awaca & Robert Colebunders

SUPPORTING MATERIAL

S2 Table: List of Species Identified from the Ituri Highlands by Date and Locality

Date (D-M-Y)	River* & Locality	Latitude/ Longitude (deg.dec)	Species & Life Stage**								
			dentulosum	*vorax*	*hargreavesi*	*bwambanum*	*alcocki*	*cervicornutum*	*Impukane/ ituriense*	*unicornutum*	*berghei*
**2015**											
15-08-15	Koda Lokpa	N02.01104 E30.90872									4 P
26-09-15	Koda Bala	N02.02499 E30.90443	5 P + 13 L								
23-10-15	Koda Gono	N02.01104 E30.90872	3 P								
19-10-15	Kuda Adrasi	N02.12322 E30.96900		1 P							
24-09-15	Kuda Adrasi	N02.12322 E30.96900	3 P + 27 L	24 P + 68 L	1 L	1 L	1 P				
20-10-15	Kuda Adrasi	N02.12322 E30.96900	7 P								
23-09-15	Kuda Ndroi	N02.10425 E30.97488		2 P			3 P				

24-09-15	Kuda<br>Ndroi	N02.10425<br>E30.97488							1 L		
19-10-15	Kuda<br>Ndroi	N02.10425<br>E30.97488	1 P + 3 L	1 NM						8 P	
26-10-15	Kuda<br>Djupacora 1	N02.11101<br>E30.96219		1 L + 1 AF		6 L					
26-09-15	Madai<br>Bala	N02.02959<br>E30.90721	8 PP			6 P				3 P	
26-09-15	Lodda<br>Zaambi	N02.04853<br>E30.91453	9 P								
Oct 2015	Lodda<br>Zaambi	N02.04862<br>E30.91464	2 P								
23-10-15	Yakpajo<br>Ndroi	N02.12459<br>E30.98039				2 P	2 P				
19-10-15	Muda<br>Adrasi	N02.09889<br>E30.9736		1 L							
24-10-15	Yadda<br>Ndeke 3	N01.97877<br>E30.91766			2 NM						
24-10-15	Gridda<br>Ndeke 3	N01.98321<br>E30.91695	3 P + 2 NM								
**2016**											
12-08-16	Ndeke	N01.97887<br>E30.91764							5 P		
15-08-16	Kuda<br>Ndroi	N02.10404<br>E30.97229		4 L							
18-08-16	Kuda<br>Kudiwek	N02.08436<br>E31.00458		20 L							
**2017**											
22-08-17	Awoo Chute<br>Djupudnik	N02.16494<br>E30.99906	1 NM + 9 P	3 NF + 1 NM							
22-08-17	Kulubu<br>(near)<br>Djuparam	N02.10439<br>E31.02308	3 AF	15 AF							

Oct-Nov 2017	Djuparam	N02.10439 E31.02308	34 AF	1 AF							
15-08-17	Kakoi Djupulungo	N02.09949 E31.03542		12 P							
18-08-17	Kakoi Djupuchelo	N02.12854 E31.04591		12 P							
16-08-17	Kuda Djupudero	N02.09819 E30.99144		60 P							
16-08-17	Alakpa Djupudero	N02.09819 E30.99144				11 P		5 P			
15-08-17	Kakoi Rima	N02.09773 E31.03268		9 L							
20-08-17	Kakoi Rima	N02.09773 E31.03268		17 P							
20-08-17	Kolubu (near) Uchudi Hill	N02.10351 E31.02260		3 AF							
21-08-17	Awoo Pont Awoo	N02.16813 E31.04783		8 P							
21-04-17	Koda Rassia	N02.00021 E30.90450	5 L + 4 P								
22-08-17	Koda Rassia Dam	N02.00021 E30.90450	14 P + 3 AF								
20-08-17	Kulubu (near Camp Uchudi)	N02.10439 E31.02308	1 AF								
19-04-17	Lodda Zaambi	N02.04862 E30.91464	7 L								
**2018**											
Feb-Mar 2018	Djuparam	N02.10439 E31.02308	37 AF	1 AF							
Mar 2018	Djuparam	N02.10439 E31.02308	64 AF	1 AF							
April 2018	Djuparam	N02.10439 E31.02308	1 AF								

05-04-18	Kulubu	N02.09487 E31.00255	12 AF								
07-04-18	Kulubu	N02.09487 E31.00255	5 AF								
08-04-18	Dabu	N02.07365 E31.02237	2 AF								

*In most cases this is the river from which samples were taken, but for appetitive female adult blackflies (AF from Human Landing Catches) this is the nearest river to the vector collection site (often situated on the bank of the river).

P=Pupae; **L=Larvae; **NF**=Neonate female adult (reared from pupae); **NM**=Neonate male adult (reared from pupae); **AF**=Appetitive female adult (from Human Landing Catch).
