# Supplementary material for: Identification of the onchocerciasis vector in the Kakoi-Koda focus of the Democratic Republic of Congo
Source: PLoS Negl Trop Dis. 2022 Nov 4;16(11):e0010684. doi: 10.1371/journal.pntd.0010684 (PMC9668120; doi:10.1371/journal.pntd.0010684)
Supplement: S3 Table — (PDF) [file pntd.0010684.s005.pdf]

**PLoSNTDs**

**Identification of the Onchocerciasis Vector in the Kakoi-Koda Focus of the Democratic Republic of Congo**

By Rory J Post, Anne Laudisoit, Christine Laemmer, Kenneth Pfarr, Achim Hoerauf, Michel Mandro, Pablo Tortosa, Yann Gomard, Tony Ukety, Thomson Lakwo, Claude Mande, Lorne Farovitch, Uche Amazigo, Didier Bakajika, David Oguttu, Naomi Awaca & Robert Colebunders

## SUPPORTING MATERIAL

# S3 Table: Crab Trapping in the Kakoi-Koda Focus in 2015-2016*

| River | Locality | Lat/long | Date Day/Month/Year | Number of Crabs | |
|---|---|---|---|---|---|
| | | | | Examined | Positive *S. neavei* |
| KUDA | NDROI | N2.10406 E30.97227 | 24/09/2015 | 18 | 0 |
| KUDA | NDROI | N2.10382 E30.97183 | 24/09/2015 | 8 | 0 |
| KUDA | ADRASI | N2.10439 E30.96429 | 25/09/2015 | 3 | 0 |
| MUDA | ADRASI | N2.09887 E30.97358 | 25/09/2015 | 3 | 0 |
| KODA | BALA | N2.0253 E30.90455 | 26/09/2015 | 0 | 0 |
| MADAI | BALA | N02.02959 E30.90721 | 26/09/2015 | 2 | 0 |
| LODDA | ZAAMBI | N2.04862 E30.91464 | 26/09/2015 | 0 | 0 |
| RANGUDDA | BALA | N2.04781 E30.91109 | 26/09/2015 | 8 | 0 |
| BAIDA | KER | N2.03369 E30.92015 | 27/09/2015 | 22 | 0 |
| BUGUDDA | NDEKE | N2.02078 E30.91233 | 27/09/2015 | 0 | 0 |
| KUDA | DJUPAKORA | N2.10339 E30.97269 | 08/06/2016 | 16 | 0 |
| KUDA | DJUPAKORA | N2.10364 E30.97332 | 08/06/2016 | 4 | 0 |
| SEPEADDA | NZERKU 3 | N1.94735 E30.9122 | 16/06/2016 | 4 | 0 |
| KUDA | DJUPAKORA | N2.10331 E30.97314 | 07/08/2016 | 0 | 0 |
| MUDA | MBESI | N2.10052 E30.97516 | 10/08/2016 | 4 | 0 |

| | | | | | |
|---|---|---|---|---|---|
| AWO | OKEBOBANDA | N2.17064 E31.04485 | 15/08/2016 | 0 | 0 |
| KAKOI | OKEBOBANDA | N2.16134 E30.05022 | 15/08/2016 | 0 | 0 |
| KUDA | SAJU | N2.10386 E30.98529 | 17/08/2016 | 3 | 0 |
| MUDA | MBESI | N2.09915 E30.97378 | 19/08/2016 | 2 | 0 |
| **Total** | | | | **97** | **0** |

*See also S4 Table for one site prospected within the focus on 23.x.2009.
