# Supplementary material for: Identification of the onchocerciasis vector in the Kakoi-Koda focus of the Democratic Republic of Congo
Source: PLoS Negl Trop Dis. 2022 Nov 4;16(11):e0010684. doi: 10.1371/journal.pntd.0010684 (PMC9668120; doi:10.1371/journal.pntd.0010684)
Supplement: S4 Table — (PDF) [file pntd.0010684.s006.pdf]

PLoSNTDs

Identification of the Onchocerciasis Vector in the Kakoi-Koda Focus of the Democratic Republic of Congo

By Rory J Post, Anne Laudisoit, Christine Laemmer, Kenneth Pfarr, Achim Hoerauf, Michel Mandro, Pablo Tortosa, Yann Gomard, Tony Ukety, Thomson Lakwo, Claude Mande, Lorne Farovitch, Uche Amazigo, Didier Bakajika, David Oguttu, Naomi Awaca & Robert Colebunders

SUPPORTING MATERIAL

S4 Table: List of Collections of Vectors from outside the Kakoi-Koda focus in 2009 & 2016

Country	Date	River	Site	Lat°N/Long°E (deg.dec)	Crabs Caught		*damnosum* larvae & pupae	Spot HLCs
					Total	*neavei* positive		
Uganda	19.x.2009	Nyadel	Nyadel village	02.4673/31.1638	22	0	0	0
		Zingili	Nyadel village	02.4694/31.2358	6	3	0	0
		Nyagak	Paidha	02.4271/30.9708	42	17	0	0
	20.x.2009	Nyagak	Akoc village	02.4464/30.9326	169	35	0	0
		Ayudi	Ayudi coffee	02.4161/30.9599	14	3	0	0
		Nyagak	Nyagale village	02.4330/30.9579	30	9	0	0
DRC	22.x.2009	Awo	Gosohi	02.2615/30.9397	0	0	0	0
		Awo	Japano village	02.1729/31.0368	2	0	0	0
		Kakoi	above Awo confluence	02.1688/31.4960	1	0	0	0
	23.x.2009	Kakoi/Tsha	Zopasonga village*	01.6643/31.5342	12	11	0	0
		Alla	Alla village	02.1794/31.1523	1	0	0	0
		Ori	Alici village	02.1835/31.1511	1	0	0	0
	24.x.2009	Omi	Aduku village	02.5719/30.6641	4	4	0	0

		Vida	Upper Nyagak	02.2293/30.8226	3	0	0	0
	25.x.2009	Grinju	Bakalu village	01.9390/30.5177	0	0	0	0
		Avida	Bunia main bridge	01.9532/30.5327	76	0	0	0
	26.x.2009	Aoda	Trib. of Shari	02.2408/30.6096	140	0	0	0
	30.iv.2016	Aru	Kakere-Mbaka bridge	02.6733/30.5424	2	0	0	0
		Omi	Kuru-kuru 2	02.6611/30.5514	3	2	0	12 *neavei*
	01.v.2016	Omi	Azabu village	02.5962/30.6339	4	3	0	0
		Omi	Aviri village	02.5718/30.6636	1	1	0	0
		Dhaka	Azabu village	02.5912/30.6167	38	1	0	0
	02.v.2016	Mi	Mbila-Lubi village	02.7714/30.6495	0	0	0	0
		Onyi	Kalia-Gombiri bridge	02.8422/30.7814	49	0	0	0
		Aru	Danger (Dibwa bridge)	02.7855/30.5880	2	0	0	0
	03.v.2016	Mi	Yomi bridge	02.7443/30.7697	48	47	0	0
		Mi	Onvoko-Malinga village	02.7218/30.7574	53	39	0	0
		Onyi	Vulo village	02.8207/30.8206	3	0	0	0
	04.v.2016	Onyi	Ombienze bridge	02.9349/30.6441	35	0	0	0
		Aru	Essebi bridge	02.9524/30.6297	13	0	0	0
		Aru	Ova bridge	03.0032/30.7476	0	0	0	0
	05.v.2016	Aru	Urua-Araba bridge	02.9525/30.7980	11	0	0	0
		Aru	Essefe village	02.8570/30.8517	3	0	0	0
		Ofo	Waro bridge	02.8860/30.8466	4	0	0	0
	06.v.2016	Mi	Ayuru 1 bridge	02.7735/30.6879	38	37	0	0
		Ake	Alivu-Nyoro bridge	02.6231/30.6231	14	9	0	0
	07.v.2016	Mi	Yomi-Onvokokakwa bridge	02.7121/30.7585	53	21	0	0

*This site is situated within the Kakoi-Koda focus.
