# Supplementary material for: Identification of the onchocerciasis vector in the Kakoi-Koda focus of the Democratic Republic of Congo
Source: PLoS Negl Trop Dis. 2022 Nov 4;16(11):e0010684. doi: 10.1371/journal.pntd.0010684 (PMC9668120; doi:10.1371/journal.pntd.0010684)
Supplement: S1 Text — (PDF) [file pntd.0010684.s007.pdf]

PLoSNTDs

Identification of the Onchocerciasis Vector in the Kakoi-Koda Focus of the Democratic Republic of Congo

By Rory J Post, Anne Laudisoit, Christine Laemmer, Kenneth Pfarr, Achim Hoerauf, Michel Mandro, Pablo Tortosa, Yann Gomard, Tony Ukety, Thomson Lakwo, Claude Mande, Lorne Farovitch, Uche Amazigo, Didier Bakajika, David Oguttu, Naomi Awaca & Robert Colebunders

SUPPORTING MATERIAL

S1 Text: Taxonomic description of recent *S. dentulosum* from the Kakoi-Koda onchocerciasis focus

Adult Female: Generally, with fewer hairs and scales on the head, thorax and abdomen than material of the same species from other localities. Head: Antennae mostly black except pale for first two segments (scape, pedicel) and basal part (1/3 to 2/3) of third segment. Clypeus and frons grey or black depending upon direction of light, with variable numbers of golden hairs, occasionally bare and sometimes with hairs at top of frons above the eyes. Thorax: Scutum dull or shiny black (sometimes depending upon light source) with dark brown postpronotal lobes. Scutum with a few golden hairs and scattered scales, with the hairs often present along the lateral and anterior margins (including postpronotal lobes). The prescutellar region often bare or with a few golden hairs (but without black hairs). Scutellum dark brown, often bare, but sometimes with golden hairs. Pleural membrane almost always bare. Katepisternum and antipronotal lobes bare. Proepisternum usually with dense covering of golden hairs, but with only a few hairs in some specimens. Tufts of black hairs present on the wings at the of base of costa and the stem of the radius. Arculus light brown. Basal tooth of claws small but distinct (usually similar to Freeman & de Meillon [1] Fig 41b). Foreleg with coxa and trochanter dark, femur yellow, tibia basally yellow but distally black, and tarsi black. Mid-leg with dark coxa and yellow/brown trochanters, femur yellow, tibia yellow basally and black distally, tarsi black. Hind leg with dark coxa and yellow trochanter, femur, tibia and basitarsus yellow basally and black distally, and other tarsi black. Abdomen: Dorsum mostly bare and black (shiny or matt depending upon direction of light), but usually some golden hairs on T2, and the sides and posterior margin of T7 and T8. Ventrum usually with a thick covering of golden hairs. Gonopophyses and paraprocts as described by Crosskey [2]. By comparison with recent specimens, historical specimens from the rivers Djuda and Tse (outside the Ituri-Albert sub-focus) were quite variable in the general abundance of hairs, but all specimens had some hairs on the pleural membrane, and the basal tooth of the claw was usually very small (like Freeman and de Meillon [1] fig 41c).

Adult Male: Only four specimens of males were identified, having been reared *en-masse* from pupae collected from Awoo Atwa on 22.viii.17. They showed striking variation in the density and distribution of hairs (with one specimen much more hairy and darker than the others). Head: Antennae similar to females. Frons with long golden hairs, and sometimes along the top of the head above the eyes. Thorax: Disc of scutum matt black with golden hairs towards the anterior margin (including postpronotal lobes), along the sides and

towards the posterior margin (including the prescutellar region), but the dorsum with only a few scattered scales or an abundance of golden hairs. Postpronotal lobes brown or black, and scutellum brown with long upstanding golden hairs. Pleural membrane with or without golden hairs. Katepisternum bare. Wings with tufts of black hairs at base of costa and at stem of vein R. Arculus brown or black. Legs with similar colour pattern to females, but darker and no yellow on the hind basitarsus which was expanded (as described by Freeman & de Meillon [1] Fig 43f). Abdomen: Dorsum black with grey patches laterally and ventrum lighter, all with light covering of golden scales or short hairs, and some specimens with long golden and black hairs laterally on T3,4,5 & 7. Ventral plate, styles and coxites as described by Freeman & de Meillon [1] (Figs 43g,h,I - although the styles may be slightly longer in specimens from Ituri-Albert focus).

Pupa: The pupa was generally similar to that described by Freeman and de Meillon [1] except that the number of pupal gill filaments was usually 15 gill filaments, instead of 14 (made up of seven pairs). In all gills examined with 15 filaments the additional one was the result of the posterior inner 'pair' being a triplet. Of 24 specimens examined for both pupal gills, 21 had 15 filaments on both sides, one had 14 on both sides, two had 15 on one side and 14 on the other, one had 15 on one side and 16 on the other (an additional inner 'pair' was manifest as a triplet). In addition, a few specimens had stubs (very short filaments), for example one damaged specimen (with only one gill) had the basic 14 filaments with the a 15th filament in the normal place but reduced to a stub. A gill from another specimen had 15 filaments, but the triplet had the stub of a sixteenth filament. The apices of the gill filaments (but not stubs) were blackened and pointed, and the surface covered with dark tubercles arranged in rows as described by Freeman and de Meillon [1] (Fig 41l). Dorsum of the thorax (especially near the base of the gill) was covered fairly densely with round tubercles. The cocoon was close-woven and shoe-shaped as described by Freeman and de Meillon [1] (Fig 41m), except that the lower lip of the neck was noticeably longer. Historical specimens examined from the rivers Djuda and Tse all had gills with 15 respiratory filaments, except one specimen which had 14 on both sides.

Last Instar Larva: Generally, similar to the description by Crosskey [3], but smaller (length 7-9 mm) and lighter in colouration. Head: Capsule generally evenly suffused light yellowish brown. Markings on the cephalic apotome present, but only very slightly darker than background colour and indistinct, and similarly for the lateral eyebrow marking. Cephalic apotome darker towards the posterior margin. Other head markings largely absent. Postgenal cleft as described by Crosskey [3], but usually subtriangular. Hypostomium as per Crosskey [3], but with circa 12 hypostomial setae in each row. Thorax: Generally light brown. Proleg circlet with 35-40 rows with circa 7-9 hooklets per row. The respiratory histoblast similar to that described by Crosskey [3] (Fig 187), but often not so curled at tips of filaments. Abdomen: Generally light brown, sometimes creamy white towards posterior ventrum. Anal sclerite as described by Crosskey [3] (Fig 167), and rectal gills with 8-10 finger-like lobules per lobe. Maxillary palp circa 2.7 to 3 times longer than width at base. Mandible as per Crosskey [3]. Cephalic Fan with circa 44 rays.

References

[1] Freeman P, de Meillon B. Simuliidae of the Ethiopian region. London: British Museum (Natural History); 1953.

[2] Crosskey RW. A re-classification of the Simuliidae (Diptera) of Africa and its islands. Bull. BM(NH) Ent. Supplement. 1969;14:1-195.
[3] Crosskey RW. A Taxonomic Study of the Larvae of West African Simuliidae (Diptera: Nematocera) with comments on the Morphology of the Larval Black-Fly Head. Bull. BM(NH) Ent. 1960;10(1):1-74.
