# Supplementary material for: Identification of the onchocerciasis vector in the Kakoi-Koda focus of the Democratic Republic of Congo
Source: PLoS Negl Trop Dis. 2022 Nov 4;16(11):e0010684. doi: 10.1371/journal.pntd.0010684 (PMC9668120; doi:10.1371/journal.pntd.0010684)
Supplement: Attachment — Submitted filename: Response reviewers 10-10-2022.docx [file pntd.0010684.s008.docx]

**REPLY TO REFEREES’ COMMENTS**

We are very pleased with the positive responses from the two referees, and would like to thank them for their kind words. Most of their criticisms were mild and we have tried to make the appropriate changes, except in a few cases, including one rather large case. We have listed them below in the same order they were sent to us. Please note that the line numbers have changed slightly between the reviewed manuscript and the resubmission because of the textual changes requested by the reviewers and because of the reformatting of the in-text citations and references to conform with the Vancouver system. Also the line numbers are different in the revised “Version for Publication” and version “Showing Changes for Editor” because the latter shows all text, whether added or deleted. The line numbers mentioned below are those shown in the version “Showing Changes for Editor”.

**Under ‘Methods’**

Reviewer 1 requested ‘a statement … as to the trapping frequency and specifically when crabs were collected within the years 2009, 2016 and 2017 and 2018”. What he/she requested was perfectly reasonable, but was the result of a minor error in the text, which was our fault. Crab trapping was carried out in 2009, 2015 and 2016, and the full collection data are presented in Supporting Material Tables S3 and S4 (as mentioned in the Results section). We have corrected the text (Lines 437-438), and taken the opportunity to add a small footnote under each of the Tables S3 and S4 to clarify the origin of one important sample.

**Under ‘Results’**

Reviewer 1 was concerned about our use of the phrase ‘seemed to have’. This issue is actually referring back to the Methods section, and we have changed the text to read ‘had’ in Lines 466 and 467, which is appropriate for the specimens in the preliminary examination, although later in the research program we found a small number of exceptions which we describe in the results section.

Reviewer 1 was also concerned about our assertion that the population of *S. dentulosum* in the Kakoi-Koda focus had an unarmed cibarium, without actually dissecting specimens ourselves. We have not dissected our few remaining adult female specimens to confirm our statement because we do not want to destroy our few remaining intact specimens before submitting them to a museum where they will act as voucher specimens which can be used by other researchers in the future to verify our taxonomic conclusions. However, we feel that our assertion is reasonable, and has a very high probability of being correct. The Crosskey reference is not just a reference for an unarmed cibarium in *S. dentulosum*. The reference surveys all African blackflies, and makes it clear that all species of the subgenus *Anasolen* (to which *dentulosum* belongs) have unarmed cibaria, and in fact there is not a single blackfly species of any subgenus in tropical Africa with an armed cibarium. Armed cibaria are known from only a small number of South American species, and have not been found in any other zoogeographical region anywhere in the whole World. Whilst it was perfectly reasonable for the referee to question our assertion (in the light of having done no dissections), we feel that readers who want to follow up our reasons will find the answer easily enough in our reference. From this point of view, we have made no change to the text.

**Under ‘Conclusions’**

Reviewer 1 was concerned about ambiguity in the Abstract and Author Summary about “becoming vectors”. However, there is a problem of space. The maximum allowed number of words is 300 and 200 words for the Abstract and Author Summary respectively. The current word counts are 298 and 196. So, we cannot simply add further explanation without cutting something out. Our paper attempts to describe and make sense of a rather complicated situation and it is difficult to find something which we can simply remove. In any case, we agree with Reviewer 1 that either scenario (which he/she describes very well) are entirely possible, and we cannot distinguish between them. That is why we purposely chose a wording that left the precise mechanism open. What would be the point in exploring these two subtly different mechanisms in the Abstract and Author Summary? So we chose not to do that, but to explore (or at least mention) them in the Discussion section of the paper. For these reasons we have not made any changes.

Reviewer 1 was also concerned about our assertion that *S. dentulosum* is a vector given the rather limited number of specimens which we examined and the use of a molecular method for detecting parasite DNA. We understand his/her concerns and so we have added (as he/she suggested) ‘probable’ to the Conclusions/Significance section of the Abstract (Line 77), and ‘probable’ to the section on ‘the origin and history of the Kakoi-Koda focus’ in the Discussion section (Line 896 – also deleting ‘apparent’ for good English).

**Under ‘Editorial and Data Presentation Modifications’**

Reviewer 1 thought that “the title, emphasizing identification, seems pedestrian”. We are pleased that the reviewer recognized that this study shows something unusual (maybe exceptional) and therefore we do not entirely disagree with him/her, but this is a matter of style and we prefer a somewhat understated style (not pedestrian), rather than something more sensationalist. This reviewer said himself/herself that it was difficult to make “an iron-tight case” for *S. dentulosum*, and although we believe that it is highly probable that it is a vector, we certainly do not know whether it is an efficient vector capable of sustaining the focus in the long run, and therefore we prefer to be the more sober and less exciting title. Therefore, we have not made any changes.

Reviewer 1 asked us to change ‘man’ to ‘human’ in line 918 (now line 924). We agree, and we have made the change as indicated, but also at three other places in the text (Lines 101, 359 and 800).

Reviewer 1 asked us to change the phrase ‘sibling species’ for ‘cryptic species’ because “cryptic species has largely replaced sibling species” and they have “slightly different meanings”. We are not at all convinced that the two terms have “slightly different meanings” (and neither is Peter Adler – the doyenne of blackfly taxonomy – in his 2022 world inventory of blackflies, cited in our paper). Furthermore, using Google Scholar we searched for papers published in 2022 using “Simuliidae”, and “sibling species” or “cryptic species” as search terms. It returned 22 publications using ‘sibling species’ and 40 using ‘cryptic species’, showing that the term ‘cryptic species’ is in the majority but ‘sibling species’ has not been ‘largely replaced’. However, having made these two points, we are rather happy that the reviewer drew our attention to this point because we agree that the term ‘sibling species’ is a rather poor descriptor (with respect to its normal usage in taxonomy), and so we are happy to make the change indicated by the reviewer in lines 796, 846-847, 1014 and 1017.

Reviewer 1 also objected to the commonly-used phrase ‘onchocerciasis vector’ (and similar constructions) on the basis that vectors transmit parasites not diseases. Should we change it? When we use these phrases, we are using a figure of speech called a synecdoche macrocosm, in which something big and complicated is used to stand in for something smaller which is only part of the big complicated whole. So, we are using the term onchocerciasis to refer to the parasite in the same way that somebody might say “I was interviewed by the New York Times”, when they were interviewed by a journalist from the New York Times. This is acceptable because whilst it is strictly speaking incorrect, ‘everybody’ knows what is really meant, and nobody will be misled to believe something incorrect. Therefore, we have made no changes. By the way, the use of ‘man’ to refer to ‘humans’ (see above), is a synecdoche microcosm. In that case, we also believe that ‘everybody knows what it means’, but we were happy to change it because many people find it offensive.

Reviewer 1 indicated that “the use of the phrases ‘was found to be’, ‘was found to show’, ‘was found to have’ and similar variants throughout the manuscript makes for tedious reading and should be deleted”. We agree with the reviewer, but deleted, not replaced? Yes, sometimes deleted, but in others the reviewer is using a synecdoche microcosm, because whilst he/she wants us to delete the ‘was found to’, he/she presumably also wants us to rewrite the remaining text so that it is still a properly formed sentence. We have been through the manuscript and made changes, as requested, in lines 157, 673, 697, 713 and 782.

**Under ‘Summary and General Comments’**

Reviewer 2 wanted us to reduce the length of the introduction by about 2/3 because it “is extremely long … and reads more like an introduction to a PhD dissertation than an introduction to a research paper”. Reviewer 1 was apparently happy with the length of the introduction. Unfortunately, reviewer 2 makes no suggestions as to which parts he/she considers to be unnecessary and not contributing to the understanding of the results. It seems that the reviewer objects on the basis of length rather than content. Our paper comes to the surprising conclusion that there is a species of blackfly transmitting onchocerciasis which has never been previously thought to be a vector. It is probably too much to describe this as revolutionary, but we believe that it has important implications. Therefore, it is important to set the finding in a full and proper context, and that is what most of the introduction seeks to provide. The ecology of the area is obviously important and that means the geology is important. The ecological history is also important. The comparison with other nearby foci cannot be ignored. The historical understanding of onchocerciasis and blackfly species in Ituri is important for understanding the current day situation, but it is complicated and difficult to disentangle and riddled with inconsistent use of the same names. Should we just ignore that and let readers try to disentangle it themselves, or would it be more useful if we present them with a coherent description which they can build on in the future? We thought very carefully about what we needed to present to the reader to help the reader to understand the context in which were trying to interpret our surprising results. We do not believe that excisions can be made without damaging the reader’s understanding of our paper. Therefore, we propose not to cut the introduction without a detailed and justified list of those parts which the reviewer believes are unnecessary.

Reviewer 2 asks us to confirm whether vector collectors were offered ivermectin. Yes, they were (see lines 427-428) and some of the vector collectors are also themselves hired as ivermectin community distributors during the yearly CDTI campaigns under the PNLO.

Reviewer 2 was surprised by the high proportions (11%) of *S. dentulosum* with *O. volvulus* DNA in their heads, and asked whether these were confirmed by dissections. The short answer is no. There were no fieldworkers who had been trained in dissection, and in any case there were too few flies to divert some of them away from PCR to field dissection. Dissection would not have itself provided a reliable identification of *Onchocerca* parasites, and so parasites would have to have been preserved for later PCR. These processes demand a level of skills much higher than vector collection and river prospections. Furthermore, the molecular identifications in the laboratory were mostly carried out after the fieldwork had been completed, and by then it was too late to think about doing dissections, or at least dissections would have to have been done on preserved vectors treated with stain to reveal the parasites, which are then unsuitable for DNA. In other words, no – the only infectivity results are those from the molecular processing of specimens.

Reviewer 2 also asks whether it would be possible to calculate ATPs. We would have loved to have done this, but unfortunately, the answer is again no, because our data is not good enough. Most of the entomological fieldwork was taken up with trying to find vector breeding sites, and even vector collection sites were difficult to find (remember, we were not able to apply the fieldwork plans commonly used for *S. damnosum* s.l., based upon breeding site prospections and associated first-line villages). So, it was difficult to set up regular vector collections in a way that would allow calculation of ATPs. We were more concerned to try to map out where vector biting was occurring. By the time we were beginning to understand the situation, project time was unavailable to set up systematic and regular collections through a whole year. Please remember that the original objective for which the project was funded, was to investigate onchocerciasis-associated epilepsy, and significant time was spent surveying epilepsy, and it was not always obvious that villages with high epilepsy were situated in areas of high biting density.

**Additional Changes Resulting from Editorial Policy**

As well as the comments from the Reviewers, we were asked to change the citation of “(Lakwo and Oguttu, unpublished data)” in the Vector Background section of the Introduction. This was because it is not PLoS policy to cite unpublished work. Our solution to this has been to publish these results ourselves, and to do this we have made changes to the Vector Background section of the Introduction, the Field Collections and Other Sources of Material section of the Methods and the Field Collection of Simuliidae section of the Results.
